# *Sordaria macrospora* Sterile Mutant pro34 Is Impaired in Respiratory Complex I Assembly

**DOI:** 10.3390/jof8101015

**Published:** 2022-09-27

**Authors:** Andrea Hamann, Heinz D. Osiewacz, Ines Teichert

**Affiliations:** 1 Institut für Molekulare Biowissenschaften, Goethe-Universität Frankfurt a.M., 60438 Frankfurt, Germany; 2 Allgemeine und Molekulare Botanik, Ruhr-Universität Bochum, 44801 Bochum, Germany

**Keywords:** fruiting body formation, ascospore formation, RNA editing, mitochondrial complex I assembly, alternative oxidase (AOX), mitochondrial respiration, cytochrome c oxidase (COX)

## Abstract

The formation of fruiting bodies is a highly regulated process that requires the coordinated formation of different cell types. By analyzing developmental mutants, many developmental factors have already been identified. Yet, a complete understanding of fruiting body formation is still lacking. In this study, we analyzed developmental mutant pro34 of the filamentous ascomycete *Sordaria macrospora*. Genome sequencing revealed a deletion in the *pro34* gene encoding a putative mitochondrial complex I assembly factor homologous to *Neurospora crassa* CIA84. We show that PRO34 is required for fast vegetative growth, fruiting body and ascospore formation. The *pro34* transcript undergoes adenosine to inosine editing, a process correlated with sexual development in fruiting body-forming ascomycetes. Fluorescence microscopy and western blot analysis showed that PRO34 is a mitochondrial protein, and blue-native PAGE revealed that the pro34 mutant lacks mitochondrial complex I. Inhibitor experiments revealed that pro34 respires via complexes III and IV, but also shows induction of alternative oxidase, a shunt pathway to bypass complexes III and IV. We discuss the hypothesis that alternative oxidase is induced to prevent retrograde electron transport to complex I intermediates, thereby protecting from oxidative stress.

## 1. Introduction

Fruiting bodies are complex three-dimensional structures that are formed during sexual propagation in filamentous ascomycetes and basidiomycetes and protect the meiospores that are generated inside [1,2]. They contain distinct cell types that do not occur during vegetative growth, as described in detail for the filamentous ascomycetes *Neurospora crassa* and *Sordaria macrospora* [3,4]. How this differentiation is mediated, and which genes play a role in fruiting body formation is the topic of ongoing studies in several ascomycetes, including *Aspergillus nidulans*, *N. crassa*, *Podospora anserina* and *S. macrospora* [1].

*S. macrospora* has served as a model system for fruiting body formation since the 1950s [5,6,7,8,9]. It forms pear-shaped fruiting bodies named perithecia after seven days of growth on a nutrient-rich medium without the need for a mating partner. The lack of aerial hyphae and mitospores allows easy access to perithecia precursor structures and means that only one developmental program is carried out, making data interpretation straightforward. Several mutagenesis approaches led to over a hundred developmental mutants with defects in perithecia formation, which were sorted into different types according to the stage of the developmental block [6]. The “pro” type mutants are blocked after the formation of protoperithecia, which are spherical perithecia precursors formed after three to four days of growth. Complementation, as well as next-generation sequencing (NGS) approaches, have led to the identification of the underlying mutation in ten pro mutants so far reviewed in [5,8]. However, a considerable number of mutants remains uncharacterized.

Work on *S. macrospora* and other fungi has led to the identification of distinct developmental proteins as well as signaling cascades and complexes and transcription factors, among others, that are required for or associated with the fruiting body and ascospore formation for an overview, see [1,5]. Another process that has recently been correlated to sexual reproduction in filamentous ascomycetes is adenosine (A) to inosine (I) mRNA editing. Editing has the potential to cause amino acid changes in proteins and thereby modify or change protein functions [10,11]. Further, several studies have identified molecular factors and pathways with a high energy demand to be linked to development. For example, autophagy is required for fruiting body formation in diverse fungi, probably to maintain high energy levels and sustain the fruiting bodies from the surrounding mycelium [12,13,14,15]. Moreover, several enzymes from primary metabolism have been shown to be required for correct development [16,17,18,19], and mitochondrial respiration has been linked to fruiting body formation by the sterile phenotype of several *N. crassa* complex I mutants [20,21]. 

In this study, we aimed to identify new developmental genes important for perithecia formation in *S. macrospora* in a forward genetics approach. By sequencing the genome of sterile mutant pro34, we identified a large deletion in the *pro34* gene encoding a putative respiratory chain assembly factor. We show that PRO34 is required for fast vegetative growth, perithecia and ascospore formation. The *pro34* transcript undergoes RNA editing, leading to an amino acid codon change. Loss of the mitochondrial PRO34 leads to respiratory defects due to lack of complex I and incorrect assembly of mitochondrial supercomplexes. Feeding electrons into the respiratory chain thus might proceed via complex II or alternative complexes (i.e., alternative NADH dehydrogenases) which are commonly found in fungi [22]. Although pro34 respires via complexes III and IV of the canonical respiratory chain pathway, surprisingly, concomitant induction of the alternative oxidase (AOX) is observed. Respiration via AOX bypasses complexes III and IV, and therefore we assume that its induction is a consequence of complex I impairment and may represent a mechanism for protection against oxidative damage.

## 2. Materials and Methods

### 2.1. Strains and Growth Conditions

All *S. macrospora* strains used in this study are listed in Table S1. Unless stated otherwise, growth conditions were as described previously [23,24]. Transformation was carried out by protoplast formation as described [23], and transformants were selected on medium containing nourseothricin (50 µg/mL). Homokaryotic strains were generated by isolating ascospores from selfed perithecia for fertile strains. Crosses were set up by inoculating agar blocks of two different strains on opposite sides of a corn meal-malt medium (BMM) plate and incubation for 7–14 days in constant light at 20–25 °C. For measuring vegetative growth, strains were pre-cultured on BMM for two days, and standard inoculants were transferred to new BMM plates. The growth front was marked after one and two days, and the distance between these two marks was measured. Data are from two independent experiments with two technical replicates each. For growth tests with AOX inhibitor salicylhydroxamic acid (SHAM, Sigma S607) and complex I inhibitor rotenone (Sigma R8875), three technical replicates per strain were tested on BMM with solvent and/or inhibitor. SHAM and rotenone were dissolved in water and dimethyl sulfoxide (DMSO) to a final concentration of 450 µg/mL and 30 µM, respectively. The growth tests with paraquat (1,1′-dimethyl-4,4′-bipyridinium dichloride; Sigma 856177) were performed with one (wild type, pro34) or two biological replicates. To this end, paraquat was diluted in water to a final concentration of 20, 100, or 200 µM.

### 2.2. Generation of Plasmids

Propagation of plasmids was performed using standard laboratory protocols [25] and *Escherichia coli* XL1 Blue MRF’ [26] as a host strain. Yeast recombination [27,28] using *Saccharomyces cerevisiae* PJ69-4A [29] as a host was applied for the cloning of plasmids. All plasmids and oligonucleotides used in this study are listed in Tables S2 and S3, respectively.

For pGFP-PRO34-CE, a 2694 bp PCR fragment (2694-03/2694-04) was transformed into yeast with *Not*I-linearized pDS23 [30]. For pPRO34-GFP, a 2692 bp PCR fragment (2694-01/2694-05) was transformed into yeast with *Hin*dIII-linearized pDS23. For pPRO34-NE and pPRO34-GFP-NE, a 1084 bp PCR fragment (2694-08/2694-09) was transformed into yeast with *Hin*dIII-linearized pGFP-PRO34-CE and *Bln*I-linearized pPRO34-GFP-CE, respectively. For pPRO34-CE, a 2693 bp PCR fragment (2694-01_2/2694-04) was transformed into yeast with *Not*I- and *Hin*dIII-digested pDS23.

### 2.3. Microscopic Investigations

Fluorescence microscopy was performed using an AxioImager M.1 microscope (Zeiss) equipped with a CoolSnap HQ camera (Roper Scientific) and a SpectraX LED lamp (Lumencor). Images were captured and edited with MetaMorph (Universal Imaging). For localization of PRO34, strains were grown on BMM-covered slides [31] for two days. Mitochondria were stained with 100 µM MitoTracker orange CMTMRos (Life Technologies, Darmstadt, Germany). GFP and MitoTracker fluorescence was analyzed using Chroma filter sets (Chroma Technology Corp.) 41017 (HQ470/40, HQ525/50, Q495lp) and 49008 (HQ560/40, ET630/75m, T585lp), respectively.

Hyphal fusion was observed after two days of growth on MMS with cellophane as described using the AxioImager M.1 microscope (Zeiss) [27].

Perithecia formation was assayed on BMM plates after seven days of growth using a Stemi 2000-C stereomicroscope (Zeiss) equipped with an AxioCamERc5s digital camera (Zeiss) and AxioVision software (Zeiss). Ascospore formation was assayed after ten days of growth on BMM plates. Perithecia were cracked open and ascus rosettes were imaged on slides using the AxioImager M.1 microscope (Zeiss). Images were processed with Adobe CS4 and CS6 (Adobe Corp.).

### 2.4. DNA Preparation, Illumina Sequencing and Mapping

Mutant pro34 from the laboratory collection of General and Molecular Botany at Ruhr-University Bochum was back-crossed several times to the wild type or brown-spored fus [32] (Figure S1A). Forty sterile strains harboring the pro34 phenotype and generating brown spores (fus background) were collected from a cross of pro34 to fus. Forty black-spored fertile strains, representing the wild type, were collected from three crosses of mutants pro30, pro32, and pro34 to fus [33] (Figure S1B). Mutants pro30 and pro32 have been described elsewhere [23,33]. DNA was extracted as described previously [32], pooled for samples pro34/fus and wt_3, respectively, and subjected to 50 bp paired-end Illumina/Solexa sequencing with a HiSeq2000 at GATC Biotech (Konstanz, Germany). Cleaning of raw data, mapping to the *S. macrospora* reference genome [34,35], analysis of sequence variants, and detection of uncovered regions was performed as described [32] using the Burrows Wheeler Alignment tool [36], SAMtools [37], and custom-made Perl scripts. Genome sequencing data have been deposited at the sequence read archive (SRA; acc. no. SRX483576 and SRX502852 for pro34/fus and wild type (wt_3) [23], respectively.

### 2.5. Nucleic Acid Isolation, cDNA Synthesis and Verification of RNA Editing

RNA isolation from mycelia was performed as described before [38]. For RNA isolation from perithecia, wild type was grown for six days on BMM plates at 27 °C and in constant light. Perithecia were scraped from the plates with a spatula and immediately transferred to 4 °C 100 % ethanol. After two to five days of fixation at 4 °C, perithecia (corresponding to approx. 100 µL volume) were transferred into a tissue grinder (Radnoti) and homogenized for 30 min in 300–500 µL of a mixture of lysis buffer (0.6 M NaCl, 2% (*w*/*v*) SDS, 10 mM EDTA, 100 mM Tris-HCl, pH 8.0), phenol and chloroform:isoamyl alcohol (24:1) in a relation of 2:1:1. Phenol extraction and DNA precipitation were performed subsequently as for mycelium samples. cDNA synthesis was performed as described before [39] with the following modifications: After DNase treatment, RNA was combined with 0.5 µg oligo-dT_12-18_ primers (Invitrogen) and 0.6 µg of random hexamers (Thermo Scientific). Reverse transcription was performed with MMLV-RT (RNase H^−^, Promega) according to the manufacturer’s protocol for 1h at 42 °C. For verification of *pro34* editing, PCR was performed on cDNA using primer pair 2694-04/2694-07 and PCR products were subjected to Sanger sequencing with primer 2694-07.

### 2.6. Western Blot Analyses

Total protein extraction from *S. macrospora* and western blot analysis with an anti-GFP antibody to detect PRO34-GFP fusions were performed as described before [40] using JL-8 primary antibody (Living Colors; TaKaRa Bio Europe/Clontech) and an anti-mouse IgG horseradish peroxidase-linked secondary antibody (Cell Signaling).

For western blot analysis with superoxide dismutase (SOD) antibodies, total protein extracts were isolated based on a protocol developed for *P. anserina* [41]. Subsequently, the proteins were separated on a 12% SDS PAGE and transferred to PVDF membranes (Immobilon-FL, Millipore). Blocking and antibody incubation of blotted PVDF membranes was performed according to the LI-COR Odyssey “Western Blot Analysis” handbook. Actin was detected with an anti-actin antibody (from mouse, Novus Biologicals NB100-74340, 1:2000). Polyclonal rabbit antibodies against Cu/ZnSOD (Biomol Stressgen #SOD-100, 1:2000) and against a synthetic peptide ([Ac]-CERFLGTSEATKL[OH], New England Peptide, 1:2000) specific for *P. anserina* MnSOD PaSOD2 were utilized to detect *S. macrospora* SODs. Secondary antibodies conjugated with the infrared dyes IRDye 800CW or IRDye 680 CW (LI-COR) were used (antibody dilution 1:15,000) for detection using the “Odyssey Infrared Imaging System” (LI-COR).

Mitochondrial protein extracts from *S. macrospora* were isolated according to [42]. To obtain the post-mitochondrial fraction, briefly, the supernatant of the first centrifugation step after the glass wool filtration was used. As this fraction contains large amounts of bovine serum albumin (BSA; as component of the isolation buffer), the protein concentration could not be determined and thus the maximal possible amount (16 µL) was loaded, while 100 µg mitochondrial fraction was used for each lane. After separation of proteins on a 12% SDS polyacrylamide gel, proteins were transferred to PVDF membranes (Immobilon-FL, Millipore) using standard protocols. Blocking and antibody incubation of blotted PVDF membranes were performed according to the LI-COR Odyssey “Western Blot Analysis” handbook. A *Sauromatum guttatum* antibody raised against the SgAOX (UniProt: P22185) full-length protein (Agrisera, product code AS20 699) was used to detect *S. macrospora* AOX proteins (UniProt: F7VMK4, F7WA39) (antibody dilution 1:100). The primary antibody to detect SmPORIN was raised against full-length PaPORIN of *P. anserina* (UniProt: B2B736) (New England peptide, antibody dilution 1:5000). To detect GFP, a GFP antibody of Sigma (G6795) was used (1:10,000). Secondary antibodies conjugated with the infrared dyes IRDye 800CW or IRDye 680 CW (LI-COR) were used (antibody dilution 1:15,000) for detection with the “Odyssey Infrared Imaging System” (LI-COR).

### 2.7. BN-PAGE and “In-Gel” Activity Assay

BN-PAGE was performed according to the protocol described in detail by [43]. To this end, for each lane, 100 or 200 µg mitochondrial protein extracts were solubilized using a digitonin/protein ratio of 3:1 (*w*/*w*). Linear gradient gels (4–13%) overlaid with 3.5% stacking gels were used for the separation of the solubilized samples. The components of the respiratory chain were visualized by Coomassie blue staining and assigned according to [44]. To identify complex IV Coomassie blue staining was omitted, and the gel was incubated in the dark in 50 mM phosphate buffer (pH 7.4) containing 1 mg/mL 3,3′-diaminobenzidine, 24 U/mL catalase, 1 mg/mL cytochrome c and 75 mg/mL sucrose [45]. To detect complex I, again Coomassie blue staining was omitted. Instead, the gel was treated with 0.1 M Tris-HCl, 0.14 mM NADH, 1.0 mg/mL nitroblue tetrazolium (NBT), pH 7.4 [45] and incubated for at least 15 min in the dark.

For “in-gel” SOD activity measurements, mycelial pieces of *S. macrospora* wild type, pro34, and pro34 transformants were grown for two days on BMM covered with cellophane foil at 27 °C under constant light and subsequently under the same conditions in liquid CM [46] for additional two days. Total *S. macrospora* protein extracts were isolated as described for *P. anserina* in [41]. “In-gel” SOD activity was determined with total protein extracts on a native polyacrylamide gel according to [41] with 100 µg total protein extract. As a loading control, an identical gel was stained with Coomassie according to standard protocols.

### 2.8. Oxygen Consumption Measurement

Mycelial pieces of *S. macrospora* wild type, pro34, and pro34 transformants were grown for two days on BMM at 27 °C under constant light and subsequently under the same conditions in liquid CM [46] for additional two days. Subsequently, small pieces of mycelium (0.3 to 5 mg) were transferred into the chamber of the “OROBOROS Oxygraph-2k” (OROBOROS INSTRUMENTS) high-resolution respirometer and oxygen consumption rate was measured in liquid CM medium according to the manufacturer’s instructions. KCN was added to a final concentration of 1 mM to inhibit respiration via complex IV. Respiration via AOX was inhibited by adding SHAM (final concentration 4 mM). For each condition, at least four different mycelial pieces were analyzed. To illustrate relative oxygen consumption after the addition of the inhibitor, absolute oxygen consumption of the respective strain in the presence of KCN or SHAM was normalized to its total absolute oxygen consumption with no added inhibitors.

## 3. Results

### 3.1. Sterility in Mutant pro34 Is Caused by a Genomic Deletion in SMAC_02694

In this study, we searched for so far unknown developmental genes that control sexual development in *S. macrospora*. Therefore, we analyzed non-allelic mutants pro9, pro24, pro34 and pro42 previously generated in a large-scale mutagenesis project for *S. macrospora* [6,47]. In contrast to the wild-type strain, which generates perithecia after seven days of growth on solid medium, these mutants are unable to generate perithecia and are thus sterile. We first assessed whether the mutation in any of the four mutants was allelic to an already described mutation causing a sterile phenotype in *S. macrospora* [23,32,33,40,47,48,49,50,51,52] or a mutation in another yet uncharacterized pro mutant. Therefore, sexual crosses were set up between pro9, pro24, pro34 and pro42 and 19 further sterile mutants with the “pro” phenotype (Table S4). In such crosses, recombinant fertile perithecia are generated in the contact region between two sterile strains in case of non-allelic mutations, while perithecia are never generated in the contact region between strains with allelic mutations. For pro9, pro24 and pro42, several crosses showed no perithecia formation (Table S4), indicating that a defect in an already described developmental gene caused sterility in these mutants. In contrast, all pro34 crosses showed massive production of perithecia in the contact region; thus, the mutation underlying the sterile phenotype in pro34 had not yet been identified.

To identify this mutation, the genome of a pool of 40 brown-spored pro34/fus strains was sequenced and analyzed using a previously established pipeline [32]. A summary of the sequencing results is shown in Table S5. Mapping of Illumina reads to the *S. macrospora* wild-type reference genome version 2 [34,35] revealed no small variant with 100% penetrance in the mutant. However, searching for unmapped regions showed a 1271 bp deletion in contig 2.11, bases 718959-720229, corresponding to bases 323-1593 of the *SMAC_02694* ORF. The *SMAC_02694* ORF has a length of 2641 bp, contains one intron at position 2379-2472 and codes for a protein of 848 amino acids. We crossed the pro34 strain again to fus to characterize the progeny and to prove that the sterile phenotype co-segregates with the deletion in the *SMAC_02694* ORF. PCR analysis of ten fertile and ten sterile ascospore isolates from this cross indeed showed the deletion in all of the sterile strains, but none of the fertile strains (Figure S2). We renamed *SMAC_02694 pro34*.

### 3.2. Fertility in pro34 Is Restored by Reintroduction of the pro34 Gene

**Figure 1:**
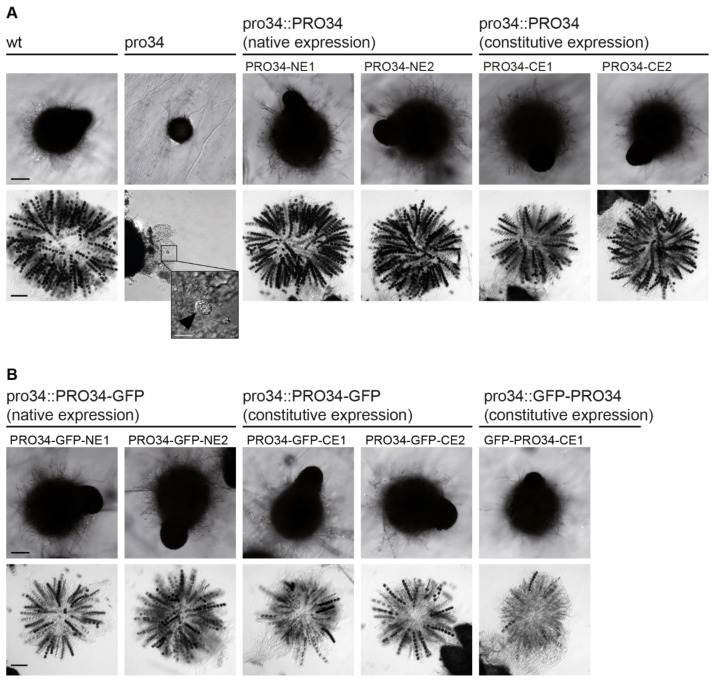
The sexual phenotype of pro34 and complemented strains. Strains were grown for seven and ten days on BMM for assaying perithecia and asci, respectively. (**A**) The phenotype of wild type, pro34 and transformants with native *pro34* gene. Wild type (wt) generates black, pear-shaped fruiting bodies with mature black ascospores, while pro34 generates immature black spherical protoperithecia that rarely contain asci that may contain immature spores (inset, arrowhead). Fruiting body and ascospore formation is restored by integration of wild-type *pro34* controlled by the native promoter (NE) and the constitutive *A. nidulans gpd* promoter (CE). The black scale bar is 100 µm; the white scale bar is 20 µm. (**B**) The phenotype of transformants with PRO34 to GFP fusions. Fruiting body formation is restored by GFP fusions, regardless of the fusion site and the promoter, but ascospore formation is not restored to wild-type levels. The black scale bar is 100 µm.

In the sterile pro34 mutant, the deletion in the *pro34* ORF leads to a frame-shift, which at the protein level results in an altered amino acid sequence from position 108 to a premature stop after 183 amino acids. To analyze the effect on fruiting body formation more closely, strains were subjected to microscopic analysis after seven days of growth. At this time point, the wild type forms pear-shaped perithecia with a diameter of 300–500 µm and a size of 2 mm (Figure 1A). Unlike the wild type, pro34 was unable to generate mature perithecia and stopped development after the formation of spherical melanized protoperithecia with a diameter of about 110 µm (Figure 1A). In contrast to wild-type perithecia, which contain asci with eight melanized ascospores each, protoperithecia of pro34 rarely contained discernable asci that sometimes harbored individual immature spores (Figure 1A, inset). To complement this phenotype, we generated plasmids carrying the complete *pro34* gene controlled by the native promoter (pPRO34-NE) or the constitutively active *A. nidulans gpd* promoter [53] (pPRO34-CE). Transformation of pro34 with each plasmid yielded fertile strains showing perithecia and ascospore formation like the wild type on the BMM fructification medium (Figure 1A). Thus, the deletion in *pro34* indeed causes the sterile phenotype of the mutant. Further, the complementation of the sexual phenotype seems to be independent of the utilized promoter. Notably, a defect in fruiting body formation is coupled to a defect in vegetative hyphal fusion in most described *S. macrospora* mutants [8]; however, pro34 is capable of vegetative hyphal fusion (Figure S3). This result indicates that PRO34 is specifically required for fruiting body and ascospore formation.

### 3.3. The pro34 Transcript Undergoes A-to-I mRNA Editing

**Figure 2:**
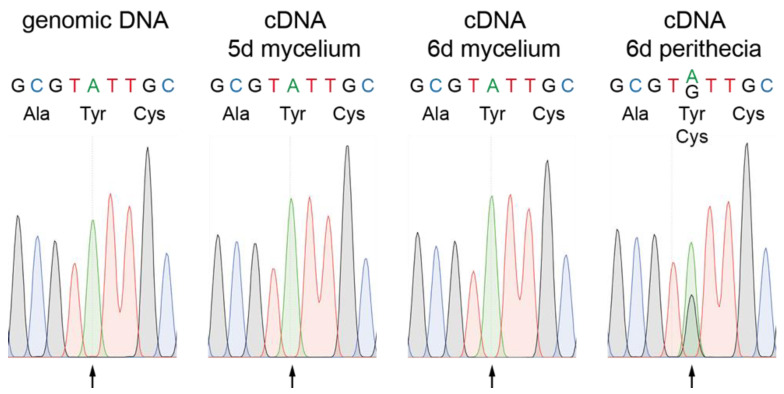
Verification of RNA editing in *pro34*. PCR fragments were generated from genomic DNA or cDNA from samples as indicated and subjected to Sanger sequencing. For each condition, chromatograms of the editing site codon and one codon up- and downstream are shown. The arrow indicates the RNA editing site at position 2562 of the *pro34* transcript. Coded amino acids are given below the DNA sequence.

Recently it was described that adenosine (A) to inosine (I) mRNA editing occurs during fruiting body formation in various filamentous ascomycetes reviewed in [10,11]. Editing often leads to amino acid variations of the encoded proteins and tends to affect genes with developmental functions, especially in ascospore formation, and genes that are differentially regulated during development [54,55,56]. We therefore searched for editing sites in the *pro34* transcript in published transcriptome datasets. Indeed, a putative A-to-I editing site was detected in the *pro34* gene in RNA-seq data from the wild type, but not from two ascospore formation mutants Δasm2 and Δasm3 and mutant Δspt3, lacking a subunit of the SAGA (Spt-Ada-Gcn5 acetyltransferase) transcriptional coactivator complex [57]. We verified the editing site in *pro34* by Sanger sequencing, where an exchange of A to guanosine (G) is indicative of an A-to-I editing event [56] (Figure 2). Genomic DNA displayed the expected A at position 2562 of the *pro34* transcript. Evidence for editing was detected in a cDNA sample from six days old perithecia, but not in cDNA samples from five- and six-day-old mycelium undergoing sexual differentiation (Figure 2). These data indicate that editing of the *pro34* transcript is correlated with fruiting body formation. In the *pro34* transcript, editing of A2562 leads to a codon change, causing an amino acid exchange of tyrosine to cysteine at position 756 of the PRO34 protein. Tyr756 resides within a globular domain predicted by the eukaryotic linear motif (ELM) resource [58], and a comparison with PRO34 homologs searched for at FungiDB [59] showed that this tyrosine is highly conserved (Figure S4). Thus, transcript editing might have a functional effect on the PRO34 protein.

### 3.4. PRO34 Localizes to Mitochondria

The putative PRO34 polypeptide is orthologous to complex I intermediate associated protein 84 (CIA84) from *N. crassa* (Figure S4) that has been described to function in mitochondrial complex I assembly [60]. PRO34 and CIA84 show 85 % identity at the amino acid level. Further, MitoFates [61] predicts a mitochondrial targeting peptide (mTP) at the N-terminus of PRO34 that is cleaved after 22 amino acids.

To determine whether or not PRO34 localizes to mitochondria in *S. macrospora*, we generated translational GFP to PRO34 fusions. Plasmids pPRO34-GFP-CE and pGFP-PRO34-CE encode C- and N-terminally tagged PRO34, respectively, expressed from the constitutive *A. nidulans gpd* promoter. Plasmid pPRO34-GFP-NE encodes C-terminally tagged PRO34 expressed from the native *pro34* promoter. All three plasmids were transformed into pro34, and transformants showed restored fruiting body formation (Figure 1B). However, ascospore formation was not restored to wild-type levels, indicating interference of the GFP tag with the function of PRO34 specifically during ascospore formation. Yet, we decided to further analyze these strains, because fruiting body formation *per se* was fully restored and the GFP tag enabled monitoring of the PRO34 protein in cellular sub-fractions by western blot analysis.

**Figure 3:**
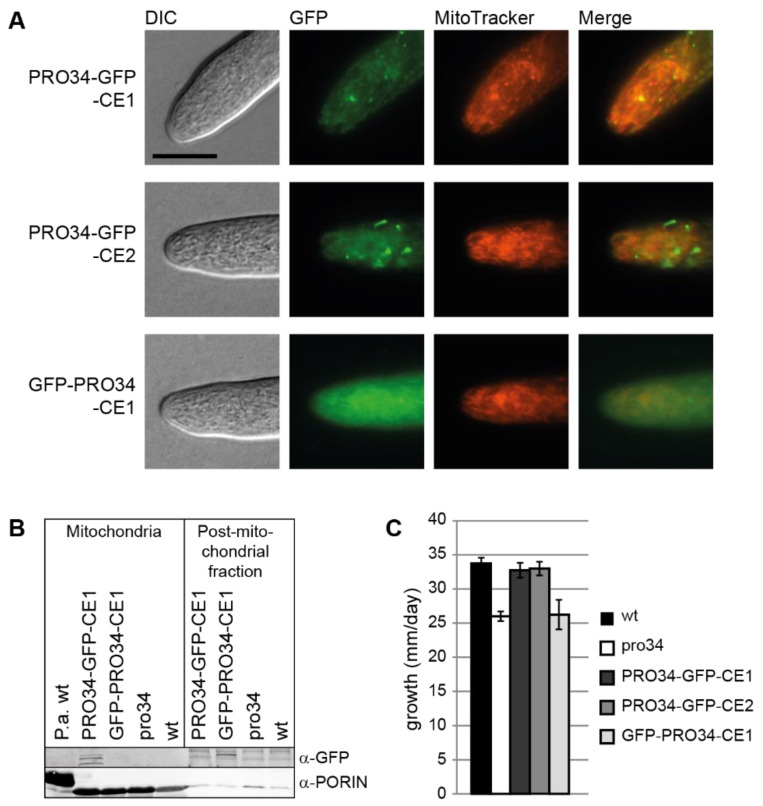
Localization of GFP-tagged PRO34 and vegetative growth. (**A**) The *pro34* gene was fused to *gfp* and expressed from the *A. nidulans gpd* promoter in the pro34 background. PRO34-GFP localizes to mitochondria, while a strain with N-terminally tagged PRO34 shows additional cytoplasmic fluorescence background. Scale bar, 10 µm. (**B**) Western blot analysis of GFP-tagged PRO34 in mitochondrial and post-mitochondrial fractions. C-terminally GFP-tagged PRO34 is detected in the mitochondrial fraction (PRO34-GFP-CE1), while N-terminally GFP-tagged PRO34 (GFP-PRO34-CE1) is not. Anti-PORIN antibody against *P. anserina* PORIN was used as a control for mitochondrial localization. *P. anserina* wild type was used as a control strain. (**C**) Vegetative growth is reduced in pro34 and in GFP-PRO34-CE1. Strains were grown on BMM and growth was measured in a 24h-interval. Data are mean and standard deviation from two biological replicates with two technical replicates each.

Western blot analysis using a GFP antibody showed that full-length GFP-tagged PRO34 with an expected size of 123.8 kDa was only detected in strains carrying C-terminally tagged PRO34, but not in strains carrying N-terminally tagged PRO34, possibly due to cleavage of the GFP (Figure S5). Three strains with distinct GFP signal were chosen for further microscopic analysis. Strains PRO34-GFP-CE1 and PRO34-GFP-CE2, carrying plasmid pPRO34-GFP-CE, showed fluorescence in mitochondria, revealed by co-staining with MitoTracker (Figure 3A). Strain GFP-PRO34-CE1 generating N-terminally tagged PRO34 showed mostly cytoplasmic fluorescence (Figure 3A). Western blot analysis of mitochondrial and post-mitochondrial fractions confirmed this localization (Figure 3B). The PRO34-GFP fusion protein was present in the mitochondrial fraction of PRO34-GFP-CE1, while the GFP-PRO34 fusion protein in GFP-PRO34-CE1 was not detected in the mitochondrial, but in the post-mitochondrial fraction. This can be explained by the cleavage of the N-terminal GFP tag during mitochondrial import as well as only partial import of this fusion protein.

Interestingly, as mentioned above, both N- and C-terminally tagged PRO34 fusion proteins were able to restore perithecia formation in the mutant (Figure 1B). However, ascospore formation was not restored to wild-type levels in all strains carrying GFP-tagged PRO34, independent of the fusion site or the expression level. Yet, differences in complementation effectiveness were evident when assaying vegetative growth. Mutant pro34 showed a reduced growth rate on BMM medium compared to the wild type. C-terminally tagged PRO34 complemented the defect, while N-terminally tagged PRO34 did not (Figure 3C). In accordance with the localization studies, these results may indicate that mitochondrial import of N-terminally tagged PRO34 is less efficient and that mitochondrial localization of PRO34 is required for complete functionality during vegetative growth.

### 3.5. Mitochondrial Respiratory Complex Assembly Is Altered in pro34

**Figure 4:**
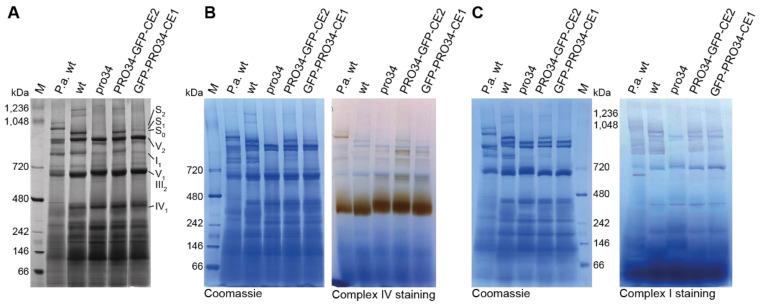
BN-PAGE and “in-gel” activity assay of mitochondrial respiratory chain complexes. For each lane, 100 (**A**,**B**) or 200 µg (**C**) mitochondrial protein extracts were solubilized using a digitonin/protein ratio of 3:1 (*w*/*w*). (**A**) The components of the respiratory chain were visualized by Coomassie blue staining and assigned according to [44]. The *Podospora anserina* wild-type strain (P.a. wt) was used as a control strain for mitochondrial complex identification. Complex V monomer (V_1_) and complex III dimer (III_2_) migrate at the same size. Complex IV staining in (**B**) and complex I staining in (**C**) were performed according to [45]. I_1_, complex I monomer; III_2_, complex III dimer; IV_1_, complex IV monomer; V_1_, complex V monomer; V_2_, complex V dimer; S_0_, supercomplex I_1_III_2_IV_0_; S_1_, supercomplex I_1_III_2_IV_1_; S_2_, supercomplex I_1_III_2_IV_2_.

The PRO34 homolog of *N. crassa*, CIA84, has a role in mitochondrial complex I assembly [60]. We therefore analyzed mitochondrial respiratory complexes in the *S. macrospora* wild type, which has not been done before, and the pro34 mutant. As a control, the *P. anserina* wild type was used, since mitochondrial respiratory complexes have been extensively studied in this model organism [41,44,62]. Complex I and complex IV staining was employed after BN-PAGE to identify the mitochondrial respiratory chain complexes as described before [44,45] (Figure 4). While *P. anserina* predominantly formed mitochondrial supercomplexes S_1_ (I_1_III_2_IV_1_) and S_2_ (I_1_III_2_IV_2_), *S. macrospora* wild type predominantly formed supercomplex S_0_ (I_1_III_2_IV_0_), but not complex IV-containing S_1_ and S_2_. However, other larger complexes with unknown compositions were present in the *S. macrospora* wild type. The pro34 mutant lacked complex I and supercomplex S_0_. This lack suggests that electrons from NADH have to be fueled into the respiratory chain by alternative proteins. Such proteins are known as internal and external alternative NADH dehydrogenases (aNADH-DHs) in other fungi, and genes encoding putative proteins with NADH dehydrogenase domains predicted by the NCBI BLAST conserved domain search [63] are also found in the genome of *S. macrospora*. Specifically, the three predicted proteins SMAC_01935, SMAC_02271, and SMAC_07176 correspond to the described aNADH-DHs NDE-2/Pa_7_5390, NDI-1/NDI1 and NDE-1/Pa_1_24200 from *N. crassa* and *P. anserina*, respectively (Figure S6) [35,64,65,66]. Complex IV was present in the pro34 mutant but seemed to migrate slower than in the wild type (Figure 4B). Strains carrying GFP-tagged PRO34 showed intermediary results. Both, PRO34-GFP-CE2 and GFP-PRO34-CE1, showed complex I in smaller amounts than in the wild type. Complex IV was also present in both strains, but like in mutant pro34, the complex migrated slower than in the wild type, supposedly indicating an altered composition.

### 3.6. Mutant pro34 Has Respiratory Defects

**Figure 5:**
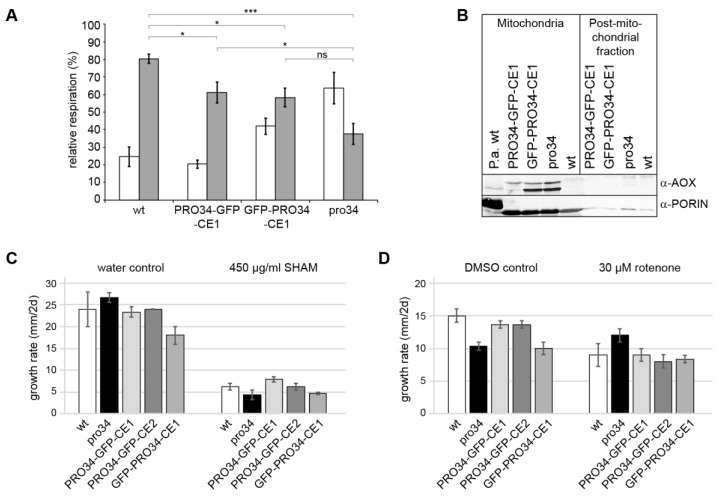
Mutant pro34 respires predominantly via the alternative oxidase pathway. (**A**) Mycelial pieces of *S. macrospora* wild type (wt), the pro34 mutant and the revertants with GFP-tagged PRO34 were used for oxygen consumption measurements. Shown is the relative respiration rate (oxygen consumption) recorded after the addition of the inhibitors potassium cyanide (KCN; white) or salicylhydroxamic acid (SHAM; gray). For each condition, at least four different mycelial pieces were analyzed: wt KCN, n = 5; wt SHAM, n = 5; pro34 KCN, n = 6; pro34 SHAM, n = 6; PRO34-GFP-CE1 KCN, n = 5; PRO34-GFP-CE1 SHAM, n = 5; GFP-PRO34-CE1 KCN, n = 6; GFP-PRO34-CE1 SHAM, n = 4; Data are mean ± SEM. * *p* < 0.05; *** *p* < 0.005; ns, not significant (Mann-Whitney, two-tailed). (**B**) Western blot analysis with an anti- alternative oxidase (AOX) antibody. Protein extracts from mitochondria and post-mitochondrial fractions were analyzed for AOX expression. Detection of the mitochondrial outer membrane protein PORIN served as a control (same as in Figure 3B). (**C**) Vegetative growth on media containing SHAM. Three technical replicates per strain were tested on BMM plates with water or SHAM. (**D**) Vegetative growth on media containing complex I inhibitor rotenone. Three technical replicates per strain were tested on BMM plates with dimethyl sulfoxide (DMSO) or rotenone solubilized in DMSO. KCN, an inhibitor of complex IV; SHAM, an inhibitor of AOX.

Since PRO34 is a mitochondrial protein and the pro34 mutants shows deficiencies in mitochondrial complex assembly, loss of the protein may lead to respiratory impairments. To assess such impairments, the wild type, pro34, PRO34-GFP-CE1 and GFP-PRO34-CE1 were subjected to respiratory measurements. Standard respiration in animals proceeds via complex I (NADH:ubiquinone oxidoreductase, inhibited by rotenone) or complex II (succinate dehydrogenase), complex III (ubiquinol:cytochrome *c* oxidoreductase), and complex IV (cytochrome *c* oxidase (COX), inhibited by potassium cyanide, KCN). In fila-mentous fungi several alternative respiratory chain components including the above-mentioned aNADH-DHs and an alternative terminal oxidase (AOX) are active under certain conditions [22,67]. In particular, the AOX (inhibited by SHAM) is well characterized. The enzyme allows to bypass of complexes III and IV at the dispense of lower proton pumping. We used KCN and SHAM to analyze the respiration of *S. macrospora* mycelium. Respiration of the wild type was strongly affected by KCN, indicating that the wild type predominantly respires via the canonical COX-dependent pathway (Figure 5A). SHAM had only a minor effect on the respiration of the wild type. In contrast, while KCN still affected pro34 respiration, SHAM affected the mutant significantly stronger than the wild type, indicating that pro34 also respires via the AOX pathway. However, although at a lower level, COX (KCN-sensitive)-dependent respiration is still observed in the mutant. Subsequently, we analyzed the phenotypes of the transformants carrying PRO34 to GFP fusion proteins. In both strains, the inhibitory effect by KCN was stronger than by SHAM, like in the wild type. In PRO34-GFP-CE1, however, inhibition by SHAM was significantly lower than in pro34 (Figure 5A), indicating that respiration is mainly COX-dependent. GFP-PRO34-CE1 showed an intermediary phenotype, and the level of SHAM inhibition was not significantly different from that of the pro34 mutant, indicating that respiration is both, COX- and AOX-dependent.

To confirm the AOX-dependent respiration in pro34, we performed a western blot analysis with an anti-AOX antibody (Figure 5B). Two genes coding for AOX proteins can be found in the *S. macrospora* genome. SMAC_08419 and SMAC_08566 correspond to AOD-3 and AOD-1 from *N. crassa* [68,69], respectively, and have a deduced size of 34 kDa and 32 kDa, respectively, after cleavage of their mTPs. The western blot (Figure 5B) indeed shows that AOX was induced in pro34 in comparison to wild type. Comparable amounts of AOX were found in strain GFP-PRO34-CE1. In PRO34-GFP-CE1, only small amounts of the AOX protein were still detectable in a western blot analysis (Figure 5B). Taken together, the introduction of a GFP-tagged PRO34 into the mutant reverted the respiration phenotype only partially, and the N-terminal tagging of PRO34 seems to either interfere stronger with its function or affect mitochondrial import efficiency.

Vegetative growth tests on a medium containing SHAM corroborated these data (Figure 5C). Growth of pro34 and GFP-PRO34-CE1 was affected on media containing SHAM. Strains PRO34-GFP-CE1 and PRO34-GFP-CE2 showed growth similar to that of the wild type, while strain GFP-PRO34-CE1 was more similar to pro34. We performed further growth tests on media containing complex I inhibitor rotenone with the same strains. Here, DMSO was used as a solvent. Although DMSO itself strongly affected growth rate, Figure 5D shows that growth of all strains except pro34 is reduced by rotenone. This result fits well with the observed lack of complex I in the mutant. PRO34-GFP-CE1, PRO34-GFP-CE2 and GFP-PRO34-CE1 did not show significantly altered growth rates in comparison to the wild type. Taken together, our data show that pro34 respires mainly via the AOX pathway and is less sensitive to complex I inhibition.

### 3.7. Mutant pro34 Shows Increased Resistance to Oxidative Stress

**Figure 6:**
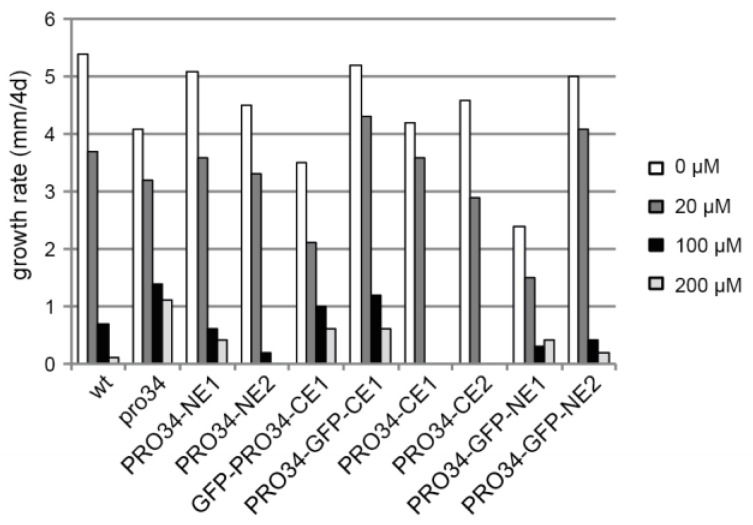
Mutant pro34 shows increased resistance to superoxide. The indicated strains were grown for four days on media with different paraquat concentrations as indicated.

A defect in mitochondrial respiration often leads to increased oxidative stress and thus affects oxidative stress resistance. To test this possibility, the mutant and the transformants were grown for four days on media containing different concentrations of paraquat, an inducer of mitochondrial superoxide production [70,71]. In contrast to wild type, mutant pro34 was able to grow on media with 200 µM paraquat (Figure 6). Transformants showed different growth phenotypes, with mainly strains carrying GFP-tagged PRO34 being able to grow on high paraquat concentrations, suggesting again that the GFP tag impairs PRO34 function.

The observed resistance to paraquat may be caused by increased levels of superoxide dismutase (SOD), the enzyme that detoxifies superoxide. We, therefore, performed a western blot analysis with antibodies raised against Cu/Zn-SOD, and PaSOD2, an ER-localized/secreted Mn-SOD [72]. The *S. macrospora* genome encodes six proteins with putative SOD function, SMAC_ 00334, SMAC_00396, SMAC_01384, SMAC_03915, SMAC_05035 and SMAC_05700. Comparison with *P. anserina* SODs indicates that SMAC_05035 may be the cytosolic Cu/Zn-SOD, SMAC_05700 the mitochondrial Mn-SOD, and SMAC_03915 an ER-localized/extracellular Mn-SOD. Western blot analysis detected one Cu/Zn-SOD and two Mn-SODs in total protein extracts, and “in-gel” SOD activity staining showed the activity of both enzyme types (Figure S7). However, SOD levels and activity were not altered in the mutant or transformed strains in comparison to the wild type. Thus, enhanced paraquat resistance is not the consequence of elevated superoxide scavenging but rather appears to be due to the lack of complex I and the identified partial respiration via AOX, which does bypass complex III, a major site of superoxide generation in the standard COX-dependent respiratory chain. In addition, respiration via AOX prevents the over-reduction of ubiquinone which might result in reverse electron transport that generates superoxide.

## 4. Discussion

In this study, we analyzed sterile mutant pro34 from *S. macrospora*. We found that the mutant has a deletion in the gene *pro34*, coding for a homolog of mitochondrial complex I chaperone CIA84 from *N. crassa* [60]. Indeed, mutant pro34 showed impairments in complex I assembly and respiration, indicating a mitochondrial function for PRO34. The mutant was further defective in vegetative growth, fruiting body and ascospore formation.

The *N. crassa* homolog of PRO34, CIA84, has been described as a mitochondrial complex I chaperone [60]. Complex I is an NADH:ubiquinone oxidoreductase complex in the inner mitochondrial membrane. Its function is to couple the electron transfer from NADH to ubiquinone with the translocation of protons across this membrane [73]. Complex I consists of two sub-complexes, a membrane arm and a peripheral arm protruding into the mitochondrial matrix, that forms an L-shaped structure [74,75]. The *N. crassa cia84* mutant accumulates the peripheral arm and a small intermediate of the membrane arm, but cannot form the complete membrane arm or the mature complex I [60]. Interestingly, mutant pro34 not only showed complex I deficiency, but also an altered migration of complex IV in a BN-PAGE and a loss of all mitochondrial supercomplexes. To our knowledge, an effect on complex IV has not been described before for a complex I protein, and in *N. crassa*, complex I was the only affected mitochondrial complex in the *cia84* mutant [60,76]. However, in the initial *N. crassa* study, mitochondrial supercomplexes were not analyzed.

In *N. crassa*, complex I has been described to be required for fruiting body and ascospore formation, but not vegetative growth [20,21,65,77,78]. The *cia84* deletion mutant was reported to show reduced vegetative growth and slightly decreased conidiation [60]. Fruiting body formation was not assayed in this study. Our data indicate that in *S. macrospora*, complex I is required for both, fast vegetative growth as well as the formation of fruiting bodies and ascospores. In general, complex I has been associated with diverse developmental processes in different organisms. Plants and fungi display several bypass options for complex I, III and IV. Nevertheless, they show an impaired redox balance when complex I is dysfunctional [79]. In humans, defects in mitochondrial complex I are associated with diseases of the central nervous system as well as pathologies of the heart and muscle, although the underlying molecular mechanisms remain mostly unclear [80]. Likewise, mitochondrial complex I assembly factors such as CIA30/NDUFAF1 [81,82] have been implicated in human pathologies [83].

Mitochondrial respiration *per se* has been linked to development before. Besides the above-mentioned *N. crassa* complex I mutants, many mutants of the aging model *P. anserina* have been described with defects in mitochondrial respiration [84]. Among these mutants, the ex1 mutant harbors a *cox1* deletion [85], thus lacking complex IV (COX) completely, while in the grisea and PaCox17::ble mutants, the delivery of copper to complex IV and thus its function is affected [86,87]. These mutants are either sterile or show a strongly reduced fertility. The developmental phenotypes of respiration mutants may be due to a high energy demand during fruiting body formation, which requires the generation of different cell types as well as the generation of meiotic progeny [1,88]. Indeed, further studies have correlated energy demand with development. For example, half of the genes encoding proteins with sugar transporter domains are differentially regulated during fruiting body development, indicating massive rearrangement of nutrient transport [35]. Further, autophagy is thought to compensate for high energy demands and to redistribute nutrients from the mycelium to the fruiting body. Accordingly, autophagy genes *atg-3* and *atg-8* from *N. crassa* are required for protoperithecia development [15], *PaAtg1*, *PaAtg8* and *PaAtg24* influence fruiting body formation, ascospore formation as well as ascospore germination in *P. anserina* [12,13,89], and *Smatg4*, *Smatg8* and *Smatg12* are required for fruiting body formation in *S. macrospora* [14,90]. Enzymes from primary metabolism also play a role in fruiting body formation, among them carbonic anhydrases that catalyze the reversible interconversion between carbon dioxide and bicarbonate, leucine biosynthesis enzyme β-isopropylmalate dehydrogenase, and ATP citrate lyase, which catalyzes the formation of acetyl-CoA and oxaloacetate from CoA [16,17,18,19]. How PRO34 functions at the molecular level during the fruiting body and ascospore formation has to be investigated in future studies.

The *pro34* transcript undergoes A-to-I RNA editing during fruiting body formation. This type of editing in nuclear protein-coding transcripts has been correlated with fruiting body formation in filamentous ascomycetes [10,11]. Several genes whose transcripts undergo A-to-I editing have been shown to function in ascospore formation, discharge and germination in different fungi. For example, deletion of major facilitator superfamily domain gene *amd1* from *Fusarium graminearum* caused defects in ascus wall formation and ascospore discharge and resulted in ascospores germinating inside the perithecia [91]. Deletion of the serine threonine kinase gene *stk-21* from *N. crassa* resulted in the generation of abnormal asci and a delay in ascospore formation. Here, we describe with *pro34* an additional gene whose transcript is affected by RNA editing and which is involved in ascospore formation. For *F. graminearum amd1* and *N. crassa stk-21* it has been shown that editing is required for proper protein function during fruiting body formation [54,91]. Further studies are necessary to analyze the effect of the single amino acid variation on PRO34 function.

In contrast to most other sterile mutants from *S. macrospora*, pro34 is able to undergo hyphal fusion. *S. macrospora* mutants with this constellation described so far are the autophagy mutants and mutant spd with the underlying *spd4* deletion, lacking a nuclear protein of unknown function [14,90,92]. Interestingly, pro34 seems to have an increased hyphal fusion rate, which might cause the massive production of perithecia in crosses to other sterile mutants, where vegetative hyphal fusion is a prerequisite to generating a competent mycelium. Although the possible mechanism underlying this observation still awaits analysis, pro34 has served as a useful tool for crossing hyphal fusion-deficient strains in the lab (IT, unpublished results).

**Figure 7:**
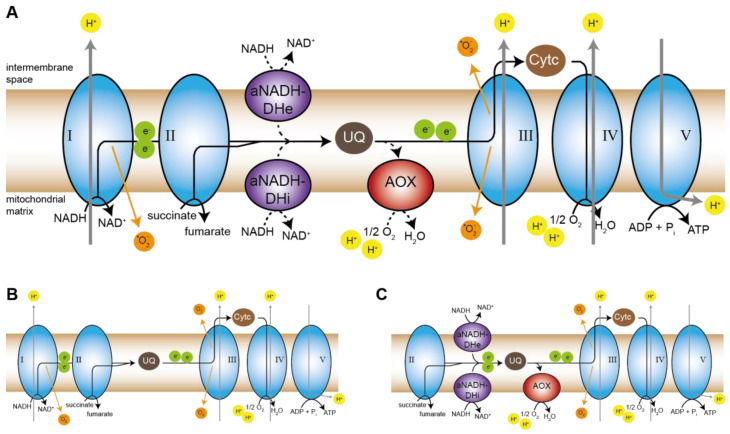
Model of respiration in wild type and pro34. (**A**) Overview of the fungal mitochondrial respiratory chain including alternative NADH dehydrogenases (aNADH-DH; e for external and i for internal enzymes in relation to the mitochondrial matrix) and alternative oxidase (AOX) bypasses. Main mitochondrial complexes are depicted in blue and numbered. The aNADH-DHs are shown in purple, AOX in red. Cytc, cytochrome c; UQ, ubiquinone. Electrons from NADH are introduced into the respiratory chain by either complex I or aNADH-DHs. Complexes I and III produce superoxide (orange). Proton (yellow) flux is depicted by gray arrows, electron (green) flux by black arrows. The route of electrons in the canonical pathway is shown by solid arrows, the route via alternative components is shown by dashed arrows. Note that only protons transported across the inner mitochondrial membrane and protons used for the generation of water by AOX and complex IV are depicted in the model. Modified from [67,84]. (**B**) Respiration in wild type follows the canonical route via complexes I through V. (**C**) Mutant pro34 lacks functional complex I and shows respiration via aNADH-DHs and complexes III and IV. Most probably, complex II and the rotenone-insensitive aNADH-DHs are used for the delivery of reducing equivalents and for feeding electrons to ubiquinone in the mutant. AOX may prevent the reshuffling of electrons to complex I intermediates and thereby prevent oxidative damage.

Coming back to the respiratory defect in pro34, the induction of AOX in the mutant is puzzling. The canonical respiration occurs via the highly conserved protein complexes complex I to V, while many fungi, like plants, can induce alternative pathways that bypass one or several protein complexes of the canonical respiratory chain (Figure 7A) [67]. In case of a lack of complex I, the canonical NADH:ubiquinone oxidoreductase, rotenone-insensitive aNADH-DHs can overtake its role in feeding electrons from NADH into the respiratory chain by reducing ubiquinone. However, they do not pump protons across the inner mitochondrial membrane [93]. Incidentally, the yeast *Saccharomyces cerevisiae* lacks complex I completely and by default employs aNADH-DHs for respiration [94,95]. In *P. anserina* aNADH-DH NDI1 has been shown to rescue a complex I-deficient mutant [66]. As mentioned above, three putative aNADH-DHs are encoded by the *S. macrospora* genome, including an ortholog of NDI1 (Figure S6) [35]. Thus, one would expect a complex I mutant to induce these enzymes and further transport electrons via complexes III and IV. Indeed, respiration of mutant pro34 is reduced by KCN-mediated inhibition of complex IV, indicating that the mutant respires via complexes III and IV. We, therefore, propose a model in which the wild type with functional complex I respires via the canonical pathway without any alternative components (Figure 7B), while pro34 uses aNADH-DHs in combination with complexes III and IV (Figure 7C). This fine-tuned special setting of the respiratory chain allows the generation of membrane potential at complexes III and VI and leads to reduced ROS and ATP production. The latter may be the key to the observed effects on growth and development.

Interestingly, we detected an induction of the AOX enzyme that displays another alternative electron transport route. AOX, found in plants and fungi, is induced to bypass complexes III and IV, and it is able to take electrons from ubiquinone to reduce oxygen to water [67] (Figure 7A,C). Like the aNADH-DH bypass, it does not generate a protonmotive force, and energy is lost as heat [93]. The induction of AOX in pro34 seems to be counter-intuitive since the mutant does respire via complexes III and IV. This kind of respiration should provide sufficient protonmotive force and consequently, pro34 is viable and even able to induce sexual development. Interestingly, also in maize plants, AOX was demonstrated to be induced upon complex I deficiency [96]. On the one hand, a possible clue is speculation raised by Maas et al. [97] who observed that in *P. anserina* neither complex III nor complex IV is required for complex I assembly. This is in sharp contrast to observations in mammals where complex III and complex IV indeed are required for complex I assembly [98,99,100,101]. It seems that in mammals, who do not have an AOX, respiratory supercomplexes which contain complexes III and IV are essential as a kind of scaffold for complex I assembly. It might be possible that in AOX-positive organisms this terminal oxidase can overtake such a function in complex I assembly assistance and therefore be induced in the pro34 mutant that cannot assemble complex I. On the other hand, AOX may prevent the reshuffling of electrons to non-functional complex I intermediates. Such intermediates may be present in mutant pro34 since they have been detected in the *N. crassa cia84* mutant [60]. AOX may thereby protect these intermediates from oxidative damage, which itself would lead to more severe phenotypes. Additional studies to validate this scenario are required. The described *pro34* mutant certainly is an excellent starting point for such an approach.

## Data Availability

Raw sequence data from sequencing mutant pro34 (pro34/fus) and wild type (wt_3) were submitted to the National Center for Biotechnology Information (NCBI) sequence read archive (accession no. SRX483576 and SRP033637).

## Supplementary Materials

The following supporting information can be downloaded at: https://www.mdpi.com/article/10.3390/jof8101015/s1, Table S1: Strains used in this study; Table S2: Plasmids used in this study; Table S3: Oligonucleotides used in this study; Table S4: Overview of crosses set up for the identification of allelic mutations; Table S5: Summary of sequence reads and small variants from genome sequencing of mutant pro34 (sample pro34/fus) and wild type (sample wt_3); Figure S1: Crossing history of strains for pro34 genome sequencing; Figure S2: Mutant pro34 harbors a deletion in gene *SMAC**_02694*; Figure S3: Mutant pro34 is able to undergo hyphal fusion; Figure S4: Amino acid alignment of PRO34 and CIA84 homologs; Figure S5: Western blot analysis of pro34 transformants carrying C- or N-terminally GFP-tagged PRO34; Figure S6: Alternative NADH dehydrogenases from *Sordaria macrospora*; Figure S7: Superoxide dismutase (SOD) levels and SOD activity. Refs. [23,30,32,33,35,40,47,48,49,50,51,52,58,59,63,64,65,66] are cited in the Supplementary Materials.
