# Supplementary material for: Sordaria macrospora Sterile Mutant pro34 Is Impaired in Respiratory Complex I Assembly
Source: J Fungi (Basel). 2022 Sep 27;8(10):1015. doi: 10.3390/jof8101015 (PMC9605262; doi:10.3390/jof8101015)
Supplement: Supplementary file 1 [file jof-08-01015-s001.zip › jof-1929836-supplementary.pdf]

Supplementary Material

Journal of Fungi

Sordaria macrospora sterile mutant pro34 is impaired in respiratory complex I assembly

Andrea Hamann 1, Heinz D. Osiewacz 1 and Ines Teichert 2,*

1 Institut für Molekulare Biowissenschaften, Goethe-Universität Frankfurt a.M., Frankfurt, Germany

2 Allgemeine und Molekulare Botanik, Ruhr-Universität Bochum, Bochum, Germany

* Correspondence: ines.teichert@rub.de

Table S1: Strains used in this study.

Strain name	Strain number	Relevant genotype and phenotype	Reference / source[a]
Wild type (wt)	S133134	Wild type (wt), fertile	AMB
pro1	S95333	pro1, sterile	[1]
pro5	S35253	pro5, sterile	AMB
pro9	S112113	pro9, sterile	AMB
pro11	S63625	pro11, sterile	[2]
pro18	S28256	pro18, sterile	AMB
pro21	S99063	pro21, sterile	AMB
pro22	S91606	pro22, sterile	[3]
pro23	S93343	pro23, sterile	[4]
pro24	S112266	pro24, sterile	AMB
pro30	S94233	pro30, sterile	[5]
pro32	S94453	pro32, sterile	[6]
pro34	S112435, S116034	pro34, sterile	AMB
pro40	S95265	pro40, sterile	[5]
pro41	S46357	pro41, sterile	[7]
pro42	S112477	pro42, sterile	AMB
pro43	S99108	pro43, sterile	AMB
pro44	S94061	pro44, sterile	[8]
Δmak1	R8501	Δ*mak1*::*hph*^{r}, sterile	[5]
Δmek1	S101796	Δ*mek1*::*hph*^{r}, sterile	[5]
Δnor1	DD574	Δ*nor1*::*hph*^{r}, sterile	[6]
Δnox1	DD27	Δ*nox1*::*hph*^{r}, sterile	[6]
Δpho1	ESP1	Δ*pho1*::*hph*^{r}, sterile	[9]
Δpro45	N161	Δ*pro45*::*hph*^{r}, sterile	[10]
PRO34-NE1<br>PRO34-NE2	R17456<br>R17474	pro34, *pro34(p)::pro34::trpC(t)*; *trpC(p)::nat1*	This study
PRO34-CE1<br>PRO34-CE2	R17644<br>R17682	pro34, *gpd(p)::pro34::trpC(t)*; *trpC(p)::nat1*	This study
PRO34-GFP-CE1<br>PRO34-GFP-CE2	R15734<br>R15786	pro34, *gpd(p)::pro34::egfp::trpC(t)*; *trpC(p)::nat1*	This study
PRO34-GFP-NE1<br>PRO34-GFP-NE2	R17492<br>R17506	pro34, *pro34(p)::pro34::egfp::trpC(t)*; *trpC(p)::nat1*	This study
GFP-PRO34-CE1	R15821	pro34, *gpd(p)::egfp::pro34:: trpC(t)*; *trpC(p)::nat1*	This study

[a] AMB, strain collection of General and Molecular Botany, Ruhr-University Bochum, Germany

Table S2: Plasmids used in this study.

Plasmid	Description	Reference
pDS23	yeast recombination vector *gpd(p)::egfp::trpC(t), amp, ura3, trpC(p)::nat1*	[11]
pGFP-PRO34-CE	*gpd(p)::egfp::pro34:: trpC(t), amp, ura3, trpC(p)::nat1*	this study
pPRO34-CE	*gpd(p)::pro34::trpC(t), amp, ura3, trpC(p)::nat1*	this study
pPRO34-GFP-CE	*gpd(p)::pro34::egfp::trpC(t), amp, ura3, trpC(p)::nat1*	this study
pPRO34-GFP-NE	*pro34(p)::pro34::egfp::trpC(t), amp, ura3, trpC(p)::nat1*	this study
pPRO34-NE	*pro34(p)::pro34::trpC(t), amp, ura3, trpC(p)::nat1*	this study

Table S3: Oligonucleotides used in this study.

**Oligonucleotide**	**sequence (5’- 3’)**
2694-01	ATGCGGTCGCATCATCTGGC
2694-01_2	TTCATCGCAGCTTGACTAACAGCTACATGCGGTCGCATCATCTGG
2694-02	TGATGGAGACGGAGTGAAGG
2694-03	ACTCTCGGCATGGACGAGCTGTACAAGATGCGGTCGCATCATCTGG
2694-04	TGATGATTTCAGTAACGTTAAGTGGATCAAGCCTCAAACACCCTG
2694-05	TGAACAGCTCCTCGCCCTTGCTCACCATAGCCTCAAACACCCTGG
2694-07	ACATCCAGCGTATCCTCACC
2694-08	TGTCGTCGTCGTCTTCGTCG
2694-09	AACGCCAGGGTTTTCCCAGTCACGACGACTCGTAACCAAGCTTGTGC

Table S4: Overview of sexual crosses set up for the identification of allelic mutations. Each cross was set up in triplicate. Numbers of perithecia in the contact region are indicated as follows: +, few perithecia; ++, several perithecia; +++, massive perithecia production. For strain references, see Table S1.

	S95333	S35253	S63625	S28256	S99063	S91606	S93343	S94233	S94453	S95265	S46357	S99108	S94061	R8501	S101796	DD27	DD574	ESP1	N161
	pro1	pro5	pro11	pro18	pro21	pro22	pro23	pro30	pro32	pro40	pro41	pro43	pro44	Δmak1	Δmek1	Δnox1	Δnor1	Δpho1	Δpro45
pro9 S112113	++	++	++	++	++	+	-	++	-	++	-	++	-	-	++	-	+	-	-
	++	++	++	+	++	+	-	++	-	++	-	++	-	-	++	-	++	-	-
	++	++	++	+	++	-	-	++	-	+	-	++	-	-	++	-	-	-	-
pro24 S112266	++	-	++	++	++	++	++	-	-	+	+++	+	++	-	+	-	++	-	-
	++	+	++	-	++	++	++	+	-	+	+++	++	++	-	-	-	++	-	-
	++	-	++	++	+	++	++	+	+	+	+++	+	++	-	++	-	++	-	-
pro34 S112435	+++	+++	+++	+++	+++	+++	+++	+++	+++	+++	+++	+++	+++	+++	+++	++	+++	++	+++
	+++	+++	+++	+++	+++	+++	+++	+++	+++	+++	+++	+++	+++	+++	+++	++	+++	++	+++
	+++	+++	+++	+++	+++	+++	+++	+++	+++	+++	+++	+++	+++	+++	+++	++	+++	++	++
pro42 S112477	++	++	++	+	++	++	++	++	++	++	+++	++	-	-	++	+	++	+	-
	++	++	++	++	++	++	++	++	++	++	+++	++	-	-	++	-	++	+	-
	++	++	++	++	++	++	++	++	++	++	+++	++	-	-	++	-	++	+	-

Table S5: Summary of sequence reads and small variants from genome sequencing of mutant pro34 (sample pro34/fus) and wild type (sample wt_3). For each sample, 40 ascospore isolates were collected. For sample pro34/fus, only brown-spored isolates with the fus background were used.

Sample	pro34/fus	wt_3
Genotype	pro34	wild type
Total number of reads	104,291,308	108,513,967
Total Mb	5319	5534
No. of reads mapped to reference genome	100,132,095	105,047,645
% of reads mapped to reference genome	96.0	96.8
Coverage	128x	134x
Number of small variants with coverage > 40 %	130	146
Number of these with 100 % penetrance	-	-

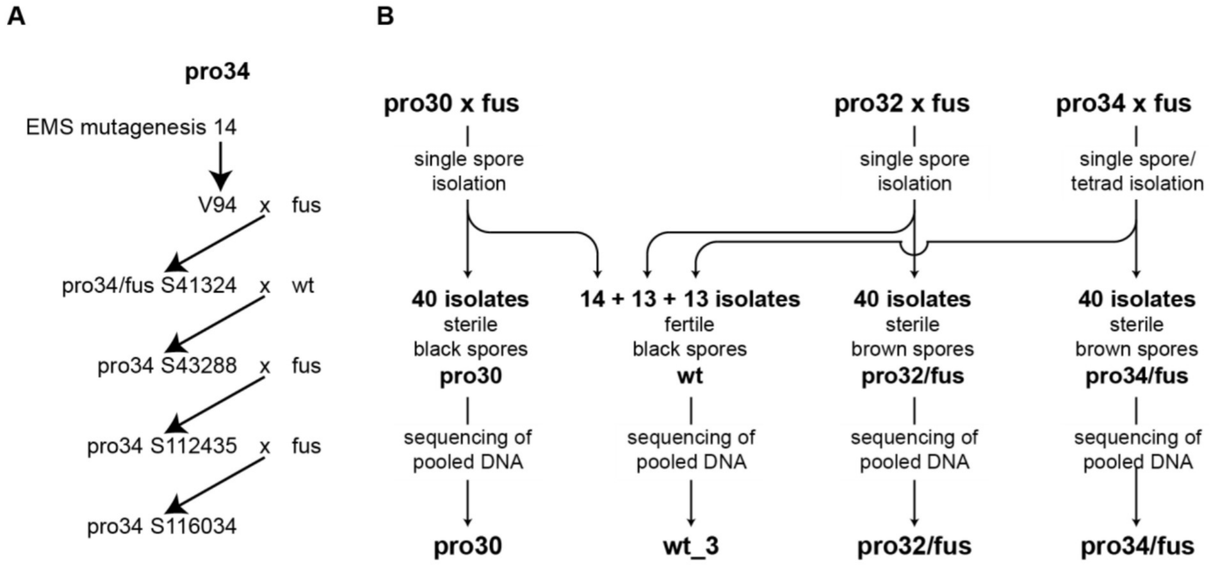

Figure S1: Crossing history of strains for pro34 genome sequencing. (A) Crossing history of mutant pro34. Mutant pro34 was generated by ethylmethanesulfate (EMS) mutagenesis and strains were crossed back to spore color mutant fus [4]. (B) Strategy for whole-genome sequencing of pooled DNA from mutants and wildtype. Mutants pro30, pro32, and pro34 were crossed to spore color mutant fus. Single spore isolates derived from black and brown ascospores were screened for fertility and color. For sample pro34/fus, 40 brown single spore isolates with a sterile phenotype were chosen. The pooled DNA from 40 single spore isolates for the pro34 genotype and the wt_3 genotype was used for sequencing. The wt_3 sample was already described [6]. Data for pro30 and pro32 have been published [5,6]. Figure S1B from [5].

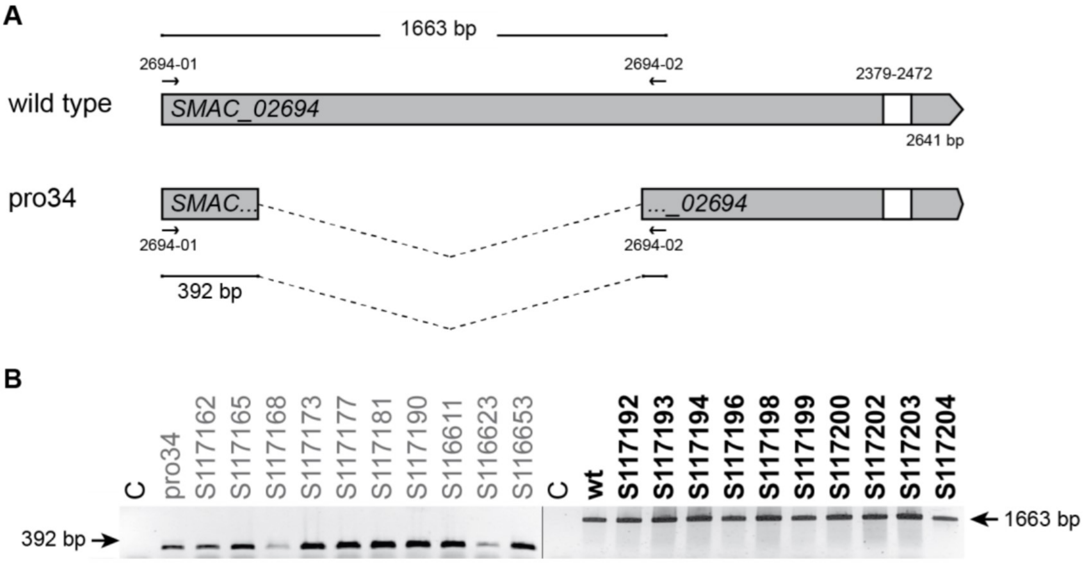

Figure S2: Mutant pro34 harbors a deletion in gene *SMAC_02694*. (A) Schematic representation of the *pro34* ORF (*SMAC_02694*) in wild type and mutant pro34. ORFs and introns are depicted by gray arrows and white boxes, respectively. Black arrows and lines denote primers and PCR fragments. Dashed lines indicate sequences deleted in the mutant. (B) The sterile phenotype co-segregates with the deletion in *pro34* in progeny of a pro34 to fus cross. PCR was performed with primers given in (A). Grey and bold letters indicate sterile and fertile strains, respectively. C, water control; wt, wild type.

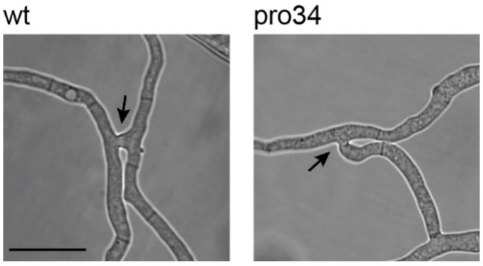

Figure S3: Mutant pro34 is able to undergo hyphal fusion. Wild type and pro34 were analyzed for hyphal fusion after two days of growth. Hyphal fusion bridges are indicated by arrows. Scale bar, 20 µm.

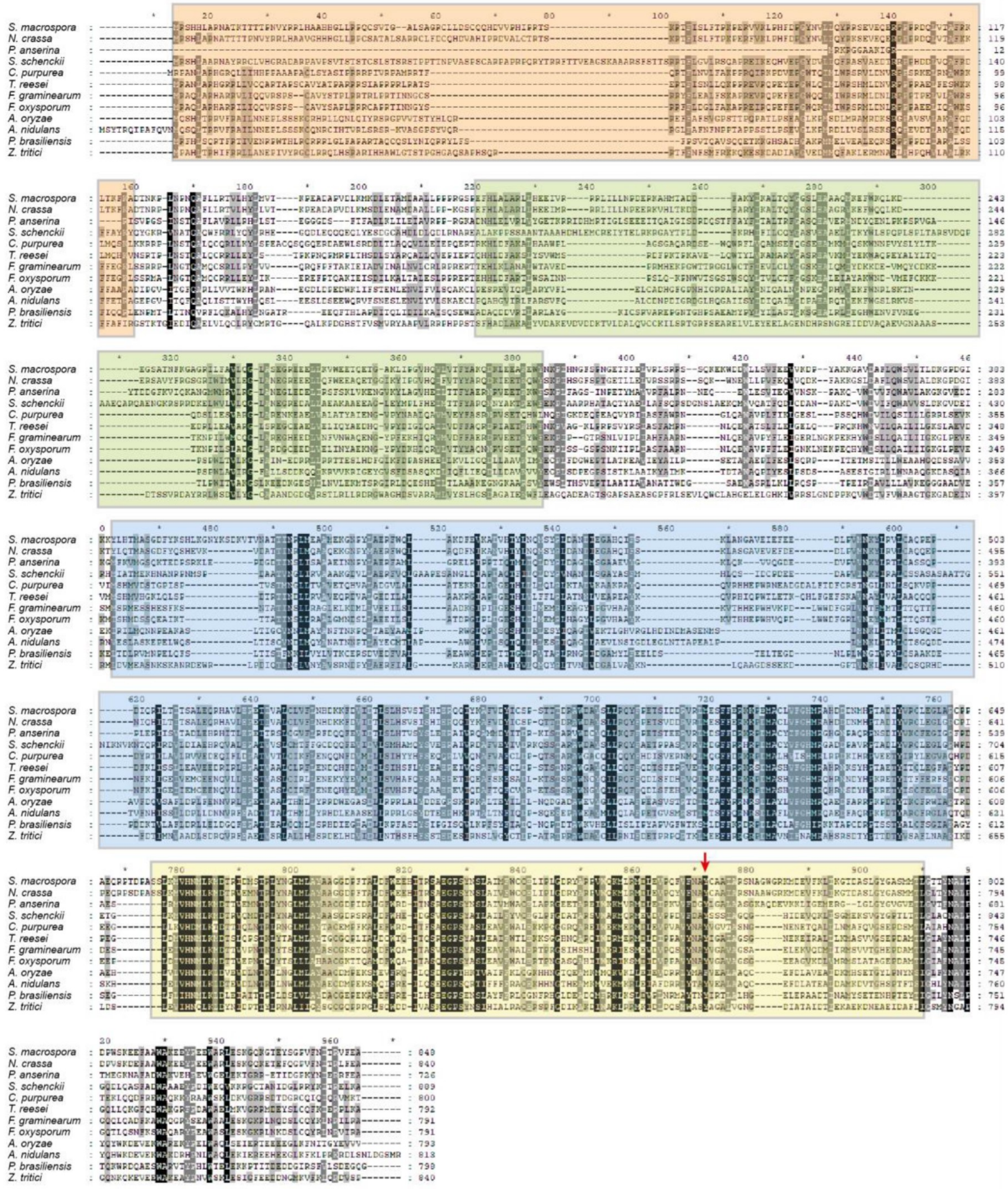

Figure S4: Amino acid alignment of PRO34 and CIA84 homologs. Protein sequences were searched for at fungidb.org [12] and can be found in the NCBI database for *Aspergillus nidulans* (CBF79406.1), *A. oryzae* (XP_001819210.1), *Claviceps purpurea* (CCE32056.1), *Fusarium graminearum* (CEF75585.1), *F. oxysporum* (EWZ36992.1), *Neurospora crassa* (CEF75585.1), *Paracoccidioides brasiliensis* (EEH47744.2), *Podospora anserina* (CDP25152.1), *Sordaria macrospora* (CCC10115.1), *Sporothrix schenckii* (KJR86726.1) and *Trichoderma reesei* (EGR45590.1), and from UniParc for *Zymoseptoria tritici* (UPI0013090055). Shaded boxes indicate four globular domains predicted for *S. macrospora* PRO34 by the eukaryotic linear motif (ELM) resource [13]. The red arrow indicates the tyrosine in the PRO34 sequence that is exchanged to cytosine by translation of edited transcripts.

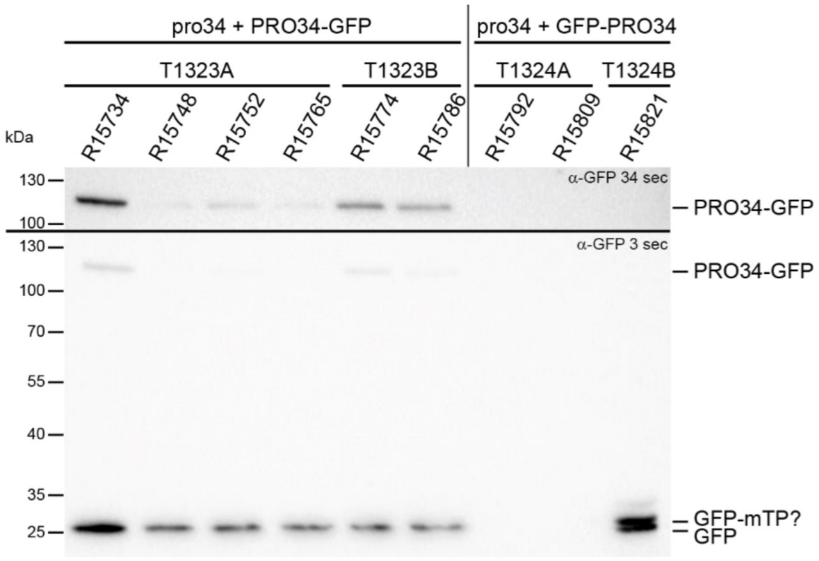

Figure S5: Western blot analysis of pro34 transformants carrying C- or N-terminally GFP-tagged PRO34. Full-length PRO34-GFP was only detectable in strains carrying C-terminally tagged PRO34. Only free GFP and a possible GFP-mTP fusion protein were detected in strain R15821, carrying N-terminally tagged PRO34. Molecular weight in kDa is given at the left. "R" designates ascospore isolates generated from the indicated primary transformants (T). Strains R15734 (PRO34-GFP-CE1), R15786 (PRO34-GFP-CE2) and R15821 (GFP-PRO34-CE1) were chosen for further analysis. mTP, mitochondrial targeting peptide.

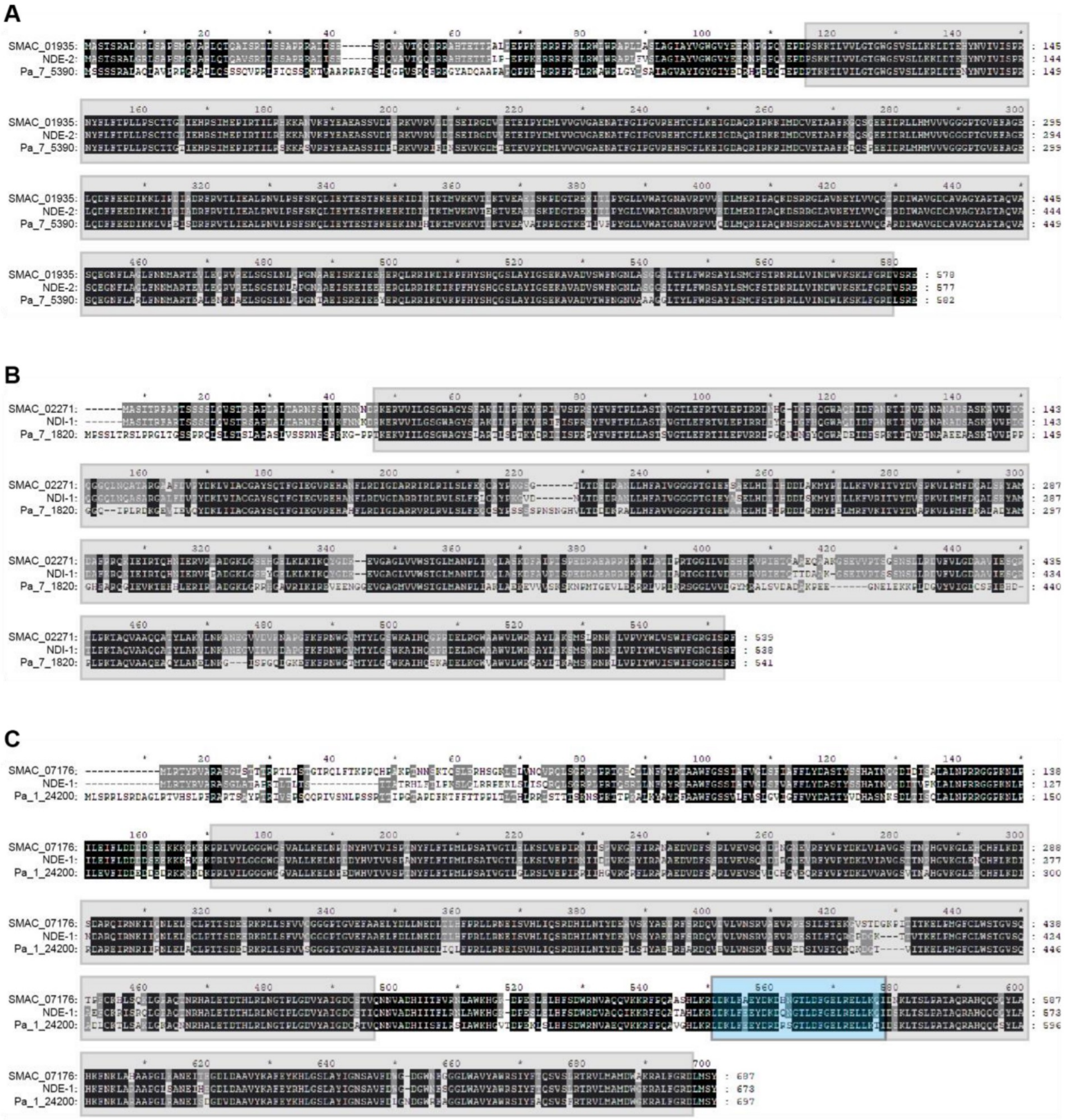

Figure S6: Alternative NADH dehydrogenases from *Sordaria macrospora*. The *S. macrospora* genome encodes three putative alternative NADH dehydrogenases (aNADH-DHs). Amino acid alignments are shown of SMAC_01935, SMAC_02271 and SMAC_07176 to their *N. crassa* and *P. anserina* orthologs [14-17]. Sequences were obtained from fungidb.org [12] for *N. crassa* and *S. macrospora*. *P. anserina* sequences can be found in the NCBI database for NDE-1 and NDE-2 orthologs. The sequence for SMAC_02271 was re-annotated according to transcriptome data. (A) Alignment of SMAC_01935 to *N. crassa* NDE-2 (NCU08980) and *P. anserina* Pa_7_5390. (B) Alignment of SMAC_02271 to *N. crassa* NDI-1 (NCU00153) and *P. anserina* NDI1 (Pa_7_1820). (C) Alignment of SMAC_07176 to *N. crassa* NDE-1 (NCU05225) and *P. anserina* Pa_1_24200. Grey boxes indicate NADH dehydrogenase domains, the blue box indicates an EF-hand domain. Domains were annotated for *S. macrospora* protein sequences according to the NCBI BLAST conserved domain search [18].

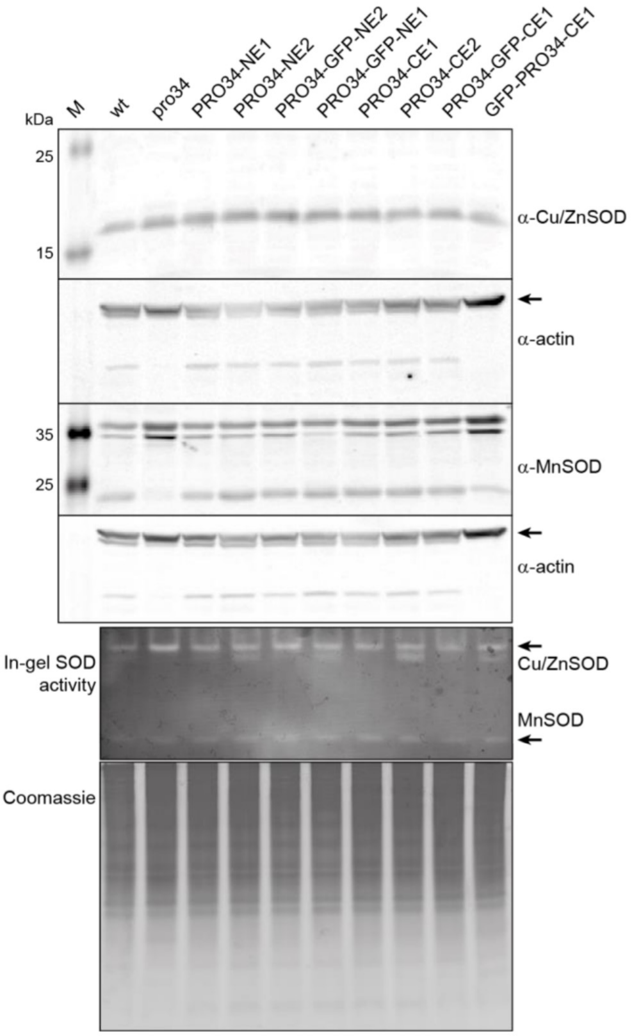

Figure S7: Superoxide dismutase (SOD) levels and SOD activity. For western blot analysis, 100 µg of total protein from the indicated strains was subjected to 12 % SDS-PAGE. Antibodies raised against Cu/ZnSOD and PaSOD2 (MnSOD) were used to detect *S. macrospora* SODs. An α-actin antibody served as control. Actin is indicated by arrows. For in-gel SOD activity staining, 100 µg total protein were subjected to 10 % native PAGE. The different SODs are indicated by arrows. Coomassie staining is shown as control.

References

1. Masloff, S.; Pöggeler, S.; Kück, U. The *pro1*(+) gene from *Sordaria macrospora* encodes a C-6 zinc finger transcription factor required for fruiting body development. *Genetics* **1999**, *152*, 191-199.
2. Pöggeler, S.; Kück, U. A WD40 repeat protein regulates fungal cell differentiation and can be replaced functionally by the mammalian homologue striatin. *Eukaryot Cell* **2004**, *3*, 232-240.
3. Bloemendal, S.; Lord, K.M.; Rech, C.; Hoff, B.; Engh, I.; Read, N.D.; Kück, U. A mutant defective in sexual development produces aseptate ascogonia. *Eukaryot Cell* **2010**, *9*, 1856-1866, doi:10.1128/EC.00186-10.
4. Nowrousian, M.; Teichert, I.; Masloff, S.; Kück, U. Whole-genome sequencing of *Sordaria macrospora* mutants identifies developmental genes. *G3 (Bethesda)* **2012**, *2*, 261-270, doi:10.1534/g3.111.001479.
5. Teichert, I.; Steffens, E.K.; Schnass, N.; Fränzel, B.; Krisp, C.; Wolters, D.A.; Kück, U. PRO40 is a scaffold protein of the cell wall integrity pathway, linking the MAP kinase module to the upstream activator protein kinase C. *PLoS Genet* **2014**, *10*, e1004582, doi:10.1371/journal.pgen.1004582.
6. Dirschnabel, D.E.; Nowrousian, M.; Cano-Dominguez, N.; Aguirre, J.; Teichert, I.; Kück, U. New insights into the roles of NADPH oxidases in sexual development and ascospore germination in *Sordaria macrospora*. *Genetics* **2014**, *196*, 729-744, doi:10.1534/genetics.113.159368.
7. Nowrousian, M.; Frank, S.; Koers, S.; Strauch, P.; Weitner, T.; Ringelberg, C.; Dunlap, J.C.; Loros, J.J.; Kück, U. The novel ER membrane protein PRO41 is essential for sexual development in the filamentous fungus *Sordaria macrospora*. *Mol Microbiol* **2007**, *64*, 923-937.
8. Schumacher, D.I.; Lütkenhaus, R.; Altegoer, F.; Teichert, I.; Kück, U.; Nowrousian, M. The transcription factor PRO44 and the histone chaperone ASF1 regulate distinct aspects of multicellular development in the filamentous fungus *Sordaria macrospora*. *BMC Genet* **2018**, *19*, 112, doi:10.1186/s12863-018-0702-z.
9. Bernhards, Y.; Pöggeler, S. The phocein homologue SmMOB3 is essential for vegetative cell fusion and sexual development in the filamentous ascomycete *Sordaria macrospora*. *Curr Genet* **2011**, *57*, 133-149, doi:10.1007/s00294-010-0333-z.
10. Nordzieke, S.; Zobel, T.; Fränzel, B.; Wolters, D.A.; Kück, U.; Teichert, I. A fungal sarcolemmal membrane-associated protein (SLMAP) homolog plays a fundamental role in development and localizes to the nuclear envelope, endoplasmic reticulum, and mitochondria. *Eukaryot Cell* **2015**, *14*, 345-358, doi:10.1128/EC.00241-14.
11. Klix, V.; Nowrousian, M.; Ringelberg, C.; Loros, J.J.; Dunlap, J.C.; Pöggeler, S. Functional characterization of MAT1-1-specific mating-type genes in the homothallic ascomycete Sordaria macrospora provides new insights into essential and nonessential sexual regulators. *Eukaryot Cell* **2010**, *9*, 894-905, doi:10.1128/EC.00019-10.
12. Basenko, E.Y.; Pulman, J.A.; Shanmugasundram, A.; Harb, O.S.; Crouch, K.; Starns, D.; Warrenfeltz, S.; Aurrecoechea, C.; Stoeckert, C.J., Jr.; Kissinger, J.C.; et al. FungiDB: An Integrated Bioinformatic Resource for Fungi and Oomycetes. *J Fungi (Basel)* **2018**, *4*, doi:10.3390/jof4010039.
13. Kumar, M.; Gouw, M.; Michael, S.; Samano-Sanchez, H.; Pancsa, R.; Glavina, J.; Diakogianni, A.; Valverde, J.A.; Bukirova, D.; Calyseva, J.; et al. ELM-the eukaryotic linear motif resource in 2020. *Nucleic Acids Res* **2020**, *48*, D296-D306, doi:10.1093/nar/gkz1030.

14. Carneiro, P.; Duarte, M.; Videira, A. The external alternative NAD(P)H dehydrogenase NDE3 is localized both in the mitochondria and in the cytoplasm of *Neurospora crassa*. *J Mol Biol* **2007**, *368*, 1114-1121, doi:10.1016/j.jmb.2007.02.080.
15. Teichert, I.; Wolff, G.; Kück, U.; Nowrousian, M. Combining laser microdissection and RNA-seq to chart the transcriptional landscape of fungal development. *BMC Genomics* **2012**, *13*, 511, doi:10.1186/1471-2164-13-511.
16. Videira, A.; Duarte, M. From NADH to ubiquinone in *Neurospora* mitochondria. *Biochim Biophys Acta* **2002**, *1555*, 187-191, doi:10.1016/s0005-2728(02)00276-1.
17. Maas, M.F.; Sellem, C.H.; Krause, F.; Dencher, N.A.; Sainsard-Chanet, A. Molecular gene therapy: overexpression of the alternative NADH dehydrogenase NDI1 restores overall physiology in a fungal model of respiratory complex I deficiency. *J Mol Biol* **2010**, *399*, 31-40, doi:10.1016/j.jmb.2010.04.015.
18. Marchler-Bauer, A.; Bo, Y.; Han, L.; He, J.; Lanczycki, C.J.; Lu, S.; Chitsaz, F.; Derbyshire, M.K.; Geer, R.C.; Gonzales, N.R.; et al. CDD/SPARCLE: functional classification of proteins via subfamily domain architectures. *Nucleic Acids Res* **2017**, *45*, D200-D203, doi:10.1093/nar/gkw1129.
